# Effect of High-Intensity Resistance Training Versus Endurance Training on Irisin and Adipomyokine Levels in Healthy Individuals: An 8-Week Interventional Study

**DOI:** 10.7759/cureus.46483

**Published:** 2023-10-04

**Authors:** P Adilakshmi, V Suganthi, K Satyanarayana Rao, K Balu Mahendran

**Affiliations:** 1 Department of Physiology, Vinayaka Missions University, Salem, IND; 2 Department of Physiology, Vinayaka Mission's Kirupananda Variyar Medical College & Hospitals, Salem, IND; 3 Department of General Medicine, Nimra Institute of Medical Sciences, Vijayawada, IND; 4 Department of Biochemistry, Siddhartha Medical College, Vijayawada, IND

**Keywords:** endurance training, high-intensity resistance training (hirt), leptin, tnf-α, interleukin-6, irisin, adipomyokine

## Abstract

Background*:* Irisin and adipomyokine are proteins secreted by the body during exercise and exhibit potential therapeutic effects for chronic disorders. Gaining insights into how high-intensity resistance training and endurance training influence irisin and adipomyokine secretion could shed light on optimizing exercise regimens for potential therapeutic applications. Such knowledge could pave the way for personalized exercise prescriptions and contribute to the development of novel treatments for chronic conditions, enhancing overall health and well-being.

Objectives: To investigate the effects of high-intensity resistance training (HIRT) and endurance training on irisin and adipomyokine levels in healthy individuals.

Methods*:* An 8-week interventional comparative study was conducted at Nimra Institute of Medical Sciences, Andhra Pradesh, India. One hundred healthy male individuals aged 21 to 35 were divided into two groups: HIRT and endurance. The HIRT group performed high-intensity resistance training, while the endurance group performed endurance training. Ethical approval was obtained, and baseline and post-intervention values of the participants were recorded and analyzed using SPSS software.

Results*:* After 8 weeks, irisin levels were significantly elevated in the HIRT group (167.39±11.27) compared to the endurance group (155.39±11.28). A positive correlation was observed between skeletal muscle and irisin levels in both the HIRT group (χ^2^-16.38; p=0.04) and the endurance group (χ^2^-18.36; p=0.01). Additionally, TNF-α (HIRT: 81.47±4.02 and Endurance: 61.19±4.00) and interleukin-6 (IL-6) (HIRT: 46.84±4.46 and Endurance: 36.15±3.89) levels significantly increased in the HIRT group. However, there was no significant change in leptin levels in either group (HIRT: 3.75±0.58 0.58 and 4.15±0.58).

Conclusion*:* The findings of this study indicate that HIRT is more effective in increasing irisin levels compared to endurance training. However, the notable elevation of IL-6 and TNF-α in the HIRT group raises concerns about potential chronic inflammation. To optimize outcomes, a combined approach, coupling HIRT and endurance training, may be beneficial. Additionally, the results emphasize the significance of skeletal muscle as a primary source of irisin secretion, implying that increased muscle contraction contributes to higher irisin release even in healthy individuals. These insights can guide exercise prescriptions and potentially enhance therapeutic strategies for chronic disorders.

## Introduction

Regular and consistent physical activity, in the form of exercise, is widely recognized as a cornerstone in the prevention and management of chronic disorders. Numerous studies have demonstrated that exercise training induces molecular modifications within the body, leading to positive health outcomes [1]. Physical activity has been shown to have medicinal properties, contributing to the amelioration of stress, anxiety, depression, hypertension, cardiovascular risks, and diabetes mellitus [2,3]. The immense therapeutic potential of exercise extends beyond physical health, positively impacting mental and emotional well-being.

The skeletal muscle, a dynamic and versatile organ, plays a pivotal role in the overall health of an individual. Apart from its well-known function in movement and locomotion, the skeletal muscle also acts as a chief excretory organ with the ability to communicate with other tissues and organs [4]. This communication occurs through the production and secretion of various proteins, the synthesis of which is highly influenced by muscle contraction. Consequently, physical inactivity and sedentary lifestyles may lead to altered skeletal muscle responses, contributing to the development of chronic diseases.

Among the proteins secreted by the skeletal muscle, irisin, a myokine, has garnered considerable attention due to its potential role in regulating blood pressure, inflammation, bone health, and brain development [5]. Irisin is derived from the proteolytic cleavage of the fibronectin type III domain-containing protein 5 (FNDC5). FNDC5 is abundantly present in both cardiac and skeletal muscles and is released into the bloodstream [6]. Upon cleavage, irisin levels increase in response to exercise, triggering the browning of white adipose tissue, which, in turn, enhances thermogenesis in muscles. This cascade of events culminates in improved obesity and glucose homeostasis, making irisin a promising therapeutic target for metabolic disorders and diseases amenable to physical exercise interventions [7].

Notably, the proinflammatory cytokine tumor necrosis factor-alpha (TNF-α) is a crucial player in the adipose tissue, playing roles in resistance to infections and cancers. It is also suggested to be involved in obesity-induced inflammation and insulin resistance [8]. Another inflammatory cytokine, interleukin-6 (IL-6), is also found in adipose tissue and is often implicated in obesity-related processes [9]. In the context of obesity, the hormone leptin is of particular interest, as it is known to efficiently decrease food intake and reduce body weight. Consequently, it was initially explored for use in the treatment of obesity [9].

The present study aims to shed light on the effects of different types of exercise training, specifically high-intensity resistance training (HIRT) and endurance training, on the levels of irisin, TNF-α, IL-6, and leptin in healthy male individuals. By comparing the impact of these exercise modalities on the levels of these proteins, the study seeks to discern their potential utility in influencing body composition and overall health in the study participants.

Understanding the dynamics of irisin and adipomyokine levels in response to distinct exercise regimens can provide valuable insights for optimizing exercise prescriptions and tailoring interventions to suit individual needs. Additionally, these findings may pave the way for novel therapeutic strategies targeting irisin and related proteins to combat chronic disorders and promote overall well-being. The significance of the skeletal muscle as a major source of irisin secretion underscores the importance of engaging in regular physical activity to harness the full potential of the body’s innate healing capabilities, even in individuals without specific health conditions. By elucidating the intricate relationship between exercise, protein secretion, and overall health, this study endeavors to contribute to the growing body of knowledge supporting the integration of exercise as a fundamental aspect of preventive and therapeutic healthcare approaches.

## Materials and methods

Study design and population

This study employed an interventional, comparative design and was conducted over an 8-week period at Nimra Institute of Medical Sciences, Andhra Pradesh. The study enrolled a total of 100 healthy male individuals aged between 21 and 35 years, individuals within this age group are generally in their prime physical condition and may respond differently to the exercise interventions compared to older or younger individuals. Notably, no female participants expressed interest in participating in the study. Participants were provided with a detailed explanation of the study objectives, procedures, potential risks, and benefits, and informed consent was obtained from all participants before commencing the study. The study protocol was approved by the Institutional Ethics Committee, ensuring compliance with ethical guidelines. Prior to the study, a power analysis was performed using G*Power software. This analysis considered a predetermined type I error rate of 0.05 and a type II error rate of 0.20, providing an 80% statistical power. The results of the analysis indicated that a total of 16 participants would be adequate to detect medium-sized “Time × Group” interaction effects (f = 0.40). To account for potential participant dropouts, a larger sample size was recruited.

Group allocation

The participants attending local gymnasiums were recruited and then by simple random method the training was assigned. The participants were divided into two groups: HIRT Group (n = 50): This group underwent an 8-week program of high-intensity resistance training. Endurance Training Group (n = 50): This group followed an 8-week endurance training program. Subjects were randomly assigned to one of the two groups.

Study procedure

Participants in both groups underwent their respective exercise regimens for 8 weeks, with five sessions per week.

HIRT Group: Participants performed 26 types of exercises, including 10 upper body exercises (bench press, cable cross-over, shoulder press, lateral raise, biceps curl, concentration curl, barbell elbow extension, and kickback) and 10 lower body exercises (squat, leg press, leg extension, seated or standing calf raise, sit-up, leg curl, back extension, sit-up, stiff leg deadlift, and abdominal crunch). Each exercise session consisted of five sets of 10-15 repetitions with a 30-second rest between repetitions, performed five times a week for 8 weeks. The exercise intensity was determined based on the target heart rate, set at 70% of 1 RM of the maximum heart rate according to the American Heart Association guidelines [10].

Endurance Training Group: Participants engaged in 30 minutes of treadmill exercise followed by 30 minutes of cycling, maintaining their heart rate at 70% of 1 RM of their maximum heart rate.

Study parameters

The study assessed the following parameters before and after the 8-week intervention period: irisin, TNF-α, IL-6, and leptin levels. Immediately within half an hour, post-exercise blood was drawn. Blood samples were collected from all participants for analysis, and anthropometric data was obtained using a portable InBody 270 compact body composition analyzer. The serum was separated from the blood samples using a centrifuge machine (3000 rpm for 3 minutes) and stored at -80°C until assayed. Serum levels of IL-6, TNF-α, and leptin were estimated using ELISA kits.

Statistical analysis

Descriptive statistical analysis was conducted using SPSS software version 21.0, with data presented as Mean±SD in tabulated and graphical forms. We used the Kolmogorov-Smirnov test to check data normality. Paired t-tests were performed to determine the significance of differences between the means of the study groups. Pearson correlation tests were conducted to assess the correlation between the study parameters. A p-value of ≤0.05 was considered statistically significant.

Ethical approval

The study obtained ethical approval from the appropriate institutional ethical committee before its initiation (Approval No. 809/NIMS/Admin/28). This ensured adherence to ethical principles, safeguarding the rights and well-being of the study participants.

## Results

Demographic features, including BMI, skeletal muscle mass, fat mass, and waist-to-hip (W/H) ratio, were similar between the two groups at baseline (Table 1).

**Table 1:** Demographic data of study participants

Study Variables	HIRT Group (n=50)	Endurance Group (n=50)
	Mean±SD	Mean±SD
BMI (kg/m²)	25.05±4.44	25.25±4.45
Skeletal mass (Before, in Kg)	28.83±3.81	28.58±3.77
Skeletal mass (After, in Kg)	29.84±3.59	28.88±3.84
Fat mass (Before, in Kg)	23.63±6.81	22.60±6.47
Fat mass (After, in Kg)	23.43±6.81	21.89±6.46
W/H ratio	0.84±0.32	0.82±0.32

The comparison of different levels in the study group is shown in Table 2.

**Table 2:** Comparison of different levels in study groups

Study groups (Irisin)	Before	After	Mean difference	p-value
HIRT group (n=50)	48.05±6.72	167.39±11.27	119.33±14.93	0.01
Endurance group (n=50)	47.07±6.56	155.39±11.28	108.32±14.74	0.02
Study groups (TNF-α)				
HIRT group (n=50)	34.03±3.49	81.47±4.02	47.43±4.65	0.01
Endurance group (n=50)	33.63±3.49	61.19±4.00	27.55±4.23	0.01
Study groups (Interleukin-6)				
HIRT group (n=50)	31.18±2.60	46.84±4.46	15.65±3.90	0.001
Endurance group (n=50)	31.68±2.60	36.15±3.89	4.46±4.81	0.694
Study groups (Leptin)				
HIRT group (n=50)	8.39±1.27	3.75±0.58	4.64±1.25	0.076
Endurance group (n=50)	7.89±1.27	4.15±0.58	3.74±1.25	0.075

Both study groups showed a significant increase in irisin levels after the 8-week intervention. Notably, the HIRT group displayed higher post-intervention irisin levels compared to the endurance group. These findings highlight the positive impact of both HIRT and endurance training on irisin levels, with potential implications for overall health and fitness.

Both study groups demonstrated a significant increase in TNF-α levels after the intervention. Notably, the HIRT group displayed higher post-intervention TNF-α levels compared to the endurance group. These findings highlight the significant impact of both HIRT and endurance training on TNF-α levels, which may have implications for inflammatory responses in the participants.

Both study groups showed significant changes in IL-6 levels after the intervention. Notably, the HIRT group exhibited higher post-intervention IL-6 levels compared to the endurance group. These findings emphasize the significant impact of HIRT on IL-6 levels, potentially influencing inflammatory responses in the participants.

Both study groups showed a decrease in leptin levels after the intervention. Notably, there were no significant differences in leptin levels between the two study groups after the intervention. These findings indicate that both HIRT and endurance training may have an impact on reducing leptin levels, which can be of clinical significance in understanding metabolic changes.

A significant correlation between skeletal muscle mass and irisin levels was observed in both exercise groups at baseline and after the intervention (Table 3).

**Table 3:** Correlation between skeletal mass and irisin levels in study groups

Study Groups	Skeletal Mass, in Kg (Baseline)	Irisin, in ng/ml (Baseline)	Pearson Correlation Test “r value”	p-value
Skeletal Mass, Kg (HIRT group)	28.83±3.81	48.05±6.72	0.261	0.04
Skeletal Mass, Kg (Endurance group)	28.58±3.77	47.07±6.56	0.259	0.04
Study groups	Skeletal Mass (After 8 weeks)	Irisin (After 8 weeks)	Pearson correlation test 'r value”	p-value
Skeletal Mass, Kg (HIRT group)	29.84±3.59	167.39±11.27	0.481	0.02
Skeletal Mass, Kg (Endurance group)	28.88±3.84	155.39±11.28	0.384	0.01

Both HIRT and endurance training lead to significant increases in irisin, TNF-α, and IL-6 levels. HIRT induced higher elevations in irisin and TNF-α levels compared to endurance training. However, leptin levels did not show significant changes after the intervention in either group. Additionally, a positive correlation between skeletal muscle mass and irisin levels highlights the role of skeletal muscle as a source of irisin secretion in response to exercise.

## Discussion

The findings demonstrate significant increases in irisin levels in both exercise groups, with a more pronounced elevation observed in the HIRT group compared to the endurance group. These results align with previous studies that have reported elevated irisin levels following exercise by Archundia-Herrera et al. and Diotaiuti et al. [11, 12].

The correlation between irisin levels and skeletal muscle mass in both study groups further supports the notion that skeletal muscle is a major source of irisin secretion. This finding is consistent with studies that have highlighted the role of skeletal muscle as an excretory organ capable of releasing various proteins in response to muscle contraction by Lecker et al., Huh et al., and Kazeminasab et al. [13-15]. Understanding the positive correlation between irisin and skeletal muscle may have implications for therapeutic strategies targeting irisin to combat obesity and related metabolic disorders. Our study observed a well-established relationship between irisin levels and skeletal muscle during different exercise types. In both the HIRT group and the endurance group, there was a statistically significant correlation between skeletal muscle mass and irisin levels at the end of the study (HIRT group: χ^2^-16.38; p=0.04, endurance group: χ^2^-18.36; p=0.01). These findings align with the study conducted by Rashid et al. (2020), where they also reported a potential role of irisin in controlling obesity and identified a correlation between irisin levels and skeletal muscle movement [16]. Moreover, our results are consistent with a study conducted by Ma et al. (2021), which highlighted that irisin is secreted by skeletal muscle and that its concentration increases significantly during high-intensity exercises [17]. Elevated irisin levels offer multiple health benefits, including reduced obesity, improved metabolic health, increased energy expenditure, cardiovascular protection, bone health, neuroprotection, antioxidant effects, mood enhancement, and anti-inflammatory properties.

Furthermore, several other studies have reported similar findings, showing that intense training leads to an increase in irisin levels, and this elevation has been associated with positive effects in reducing various health risks. These collective results support the notion that irisin plays a crucial role in the regulation of body weight and overall health, making it a potential target for therapeutic interventions against obesity and related conditions [18, 19].

Our study, along with other scientific evidence, emphasizes the significance of irisin in the context of exercise and its association with skeletal muscle. Understanding the mechanisms behind irisin’s secretion and its effects on the human body may pave the way for the development of novel strategies to combat obesity and improve overall health through exercise-based interventions. Furthermore, the potential development of recombinant irisin and its administration as a therapeutic agent holds promising prospects for targeting obesity and related health conditions.

The elevated levels of the proinflammatory cytokines TNF-α and IL-6 in both exercise groups indicate that both HIRT and endurance training can stimulate inflammatory responses. This inflammation is necessary for the repair and adaptation of the skeletal muscles to exercise stimuli. It is important to consider that the elevated levels of these markers may also be influenced by the withdrawal of blood immediately after exercise, as both markers take some time to be cleared from circulation. Additionally, the higher values may be attributed to the high inflammatory reaction of skeletal muscles during HIRT, which can further contribute to the release of these cytokines.

However, the HIRT group exhibited significantly higher levels of TNF-α and IL-6 compared to the endurance group. These results are consistent with previous research linking high-intensity exercise to increased proinflammatory cytokines [20]. The potential for chronic inflammation induced by HIRT warrants attention, as sustained inflammation can have adverse effects on overall health and may counteract some of the benefits of exercise. It is important to consider the balance between exercise intensity and inflammatory response, as excessive inflammation can lead to detrimental effects.

Interestingly, leptin levels did not show significant changes in either exercise group after the intervention. This contrasts with previous studies that have reported leptin’s role in endurance training [21]. The lack of significant change in leptin levels may be attributed to individual variability in response to exercise as well as the study’s relatively short duration. Future investigations with a more extended intervention period and a larger sample size may provide further insights into the effects of different exercises on leptin levels.

Overall, the results of this study contribute to the growing body of evidence supporting the positive effects of exercise on irisin levels and its potential therapeutic significance for metabolic disorders. The elevated levels of proinflammatory cytokines in the HIRT group raise considerations for exercise prescription and emphasize the importance of personalized exercise programs based on individual health status and goals. Combining HIRT with endurance training may offer a balanced approach to harnessing the benefits of both exercise modalities while mitigating the potential adverse effects of excessive inflammation. Moreover, adopting an anti-inflammatory diet rich in fruits, vegetables, whole grains, and omega-3 fatty acids can further complement the exercise regimen and help reduce inflammation. This integrated approach of combining exercise and anti-inflammatory foods may enhance overall health outcomes and provide a comprehensive strategy to combat inflammation and its associated health risks.

Limitations of this study include its relatively small sample size and the exclusion of female participants, which limit the generalizability of the findings. Additionally, the study’s duration may not capture long-term changes in the measured parameters. Future research should address these limitations and explore the effects of exercise on irisin and adipomyokine levels in diverse populations and over extended periods.

## Conclusions

This study provides valuable insights into the impact of different types of exercise on irisin and adipomyokine levels. HIRT demonstrated superior efficacy in increasing irisin levels compared to endurance training. The observed elevation of proinflammatory cytokines in the HIRT group highlights the need for careful exercise prescription and suggests the potential benefits of combining HIRT with endurance training for optimizing health outcomes. Understanding the intricate relationship between exercise, protein secretion, and inflammation can inform evidence-based exercise prescriptions and improve therapeutic strategies for chronic disorders. Further research in this area is warranted to advance our understanding of the therapeutic potential of exercise-induced protein responses and their application in promoting health and well-being.
